# Natural Maghemite Nanoparticles in Crude Oil Seen by Electron Paramagnetic Resonance

**DOI:** 10.1021/acsomega.5c12675

**Published:** 2026-03-05

**Authors:** Marcio S. Pessoa, Paulo Sérgio Moscon, Maristela Vicente, Maria Fatima Pereira Santos, Erico Marlon Moraes Flores, Paulo César Morais, Paulo Eduardo Narcizo de Souza, Marcos Sousa, Edson Caetano Passamani

**Affiliations:** † Department of Natural Sciences, Northern Espírito Santo University Center, Federal University of Espírito Santo 29932-540 São Mateus, ES, Brazil; ‡ Department of Chemistry, Federal University of Santa Maria 97105-900 Santa Maria, RS, Brazil; § Institute of Physics, University of Brasília, Brasilia 70910-900, Brazil; ∥ Institute of Exact Sciences and Technologies, Federal University of Jataí, Jataí 75801-615, Brazil; ⊥ Department of Physics, Federal University of Espírito Santo, Av. Fernando Ferrari, Vitória, Espírito Santo, 514 29075-910, Brazil

## Abstract

The characterization of inorganic particulate matter suspended in crude oil is crucial for understanding reservoir geochemistry, fluid provenance, and potential issues related to production infrastructure. In this regard, this work represents the first methodology that applies a physics-based model to systematically characterize natural magnetic nanoparticles (MNPs) in a diverse set of crude oils, establishing electron paramagnetic resonance (EPR) as a robust tool for magnetic speciation in complex organic fluids with a low concentration of MNPs. More specifically, it presents a comprehensive investigation into the magnetic properties of naturally occurring MNPs found in five distinct crude oil samples (A–E) from the Esprito Santo sedimentary basin. Despite the inherently low concentration of these magnetic phases, which renders them undetectable by conventional X-ray diffraction, we successfully isolated and characterized them by using a high-gradient magnetic concentration protocol, followed by EPR spectroscopy at 9.4 GHz. The EPR spectra exhibited broad, asymmetric resonance signals characteristic of randomly oriented nanoparticles with cubic-type anisotropy. Applying the Tsay model for an ensemble of Fe-oxide nanoparticles, we extracted fundamental magnetic parameters from the MNP ensemble: the peak-to-peak line width (*W*), the effective g-factor (*g* ≈ 2.08–2.15), and the anisotropy field (*H*_1_ ≈ −350 to −480Oe). The results revealed that the magnetic phase is predominantly maghemite (γ-Fe_2_O_3_). A correlation analysis between the magnetic parameters, iron concentration, and total acid number (TAN) suggests basically two distinct sources for the existence of MNPs, which are represented by Sample A, characterized by high acidity (1.81 mg of KOH·g^–1^) and iron content (8.3 μg g^–1^), exhibiting magnetic properties consistent with corrosion-derived particles, whereas Sample D, with low acidity (0.10 mg of KOH·g^–1^) and iron content (0.9 μg g^–1^), showing a signature indicative of intrinsic geogenic nanoparticles.

## Graphical abstract

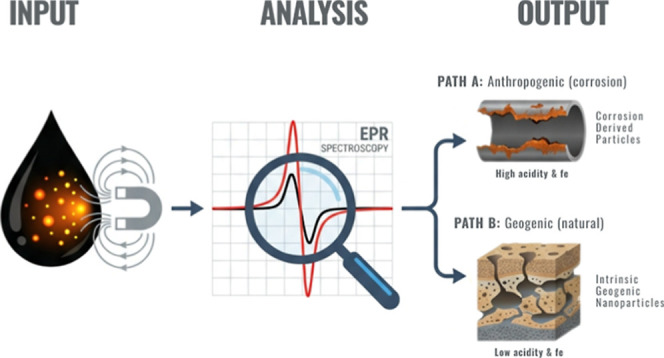

## 1 Introduction

Crude oil is a complex and multiphase colloidal system composed primarily of hydrocarbons, heteroatoms (sulfur, nitrogen, and oxygen), and trace amounts of metallo–organic complexes.^1,2^ While transition metals (e.g., vanadium and nickel) are well-documented as stable porphyrin complexes within the asphaltene fraction, iron is frequently present as suspended inorganic particulates, ranging from micron-sized scales down to the nanoscale.^3^ The presence of natural magnetic nanoparticles (MNPs) in petroleum is a subject of growing interest in the field of “Novel Magnetism” due to their potential applications in reservoir characterization, flow assurance, and enhanced oil recovery.^4^ It should be mentioned that the MNPs, typically iron oxides such as magnetite (Fe_3_O_4_) or maghemite (γ-Fe_2_O_3_), may originate from various sources: biogenic synthesis by magnetotactic bacteria,^5^ petrogenic weathering of reservoir rocks,^6^ or anthropogenic corrosion of steel production infrastructure driven by acidic oil components.^7^

Despite their significance, the detection and characterization of these MNPs present a formidable analytical challenge due to their extremely low concentrations, often in the parts-per-million (ppm) range, and the overwhelming diamagnetic signal of the hydrocarbon matrix.^8^ Conventional structural techniques like X-ray diffraction (XRD) are often insufficient, as their low volume fraction and peak broadening, associated with their nanometric crystallites, result in diffraction peaks that are strongly masked by the amorphous scattering of the organic background.^9^ Consequently, highly sensitive spectroscopic methods are required. Electron paramagnetic resonance (EPR), or ferromagnetic resonance (FMR) when applied to magnetically ordered systems, offers the sensitivity required to detect unpaired electrons and magnetic moments at trace levels.^10,11^ While previous studies have reported the presence of superparamagnetic colloids in petroleum using magnetic susceptibility and EPR,^12^ a systematic characterization using theoretical models to extract intrinsic magnetic parameters such as anisotropy fields remains scarce in the literature and would bring some additional information for the presence and sources of these iron MNPs.

In this study, we investigate five dehydrated crude oil samples (A–E) with varying physicochemical properties. To overcome the detection limits of standard spectroscopy, we employed a magnetic concentration procedure to enrich the MNP fraction. The core of this work involves the analysis of the EPR spectra using the phenomenological model proposed by Tsay et al. (1971). Originally developed to interpret the FMR spectra of lunar regolith samples containing metallic iron and oxides, the Tsaỳs model is particularly effective for analyzing polycrystalline or randomly oriented assemblies of magnetic particles with cubic anisotropy.^13,14^ By fitting the experimental spectra to this model, we determined the line width (*W*), g-factor (*g*), and anisotropy field (*H*_1_) for the magnetic phases in five crude oils. These parameters provided a magnetic fingerprint that allowed us to identify the particles as maghemite and to discuss their potential origins (corrosion versus geogenic) by correlating the magnetic data with the oil’s acidity (TAN) and elemental iron content determined by ICP-OES. This study brings additional information to the crude oil field, specifically in the identification of low concentration Fe-oxide particulates and their potential sources.

## 2 Materials and Methods

The study utilized five crude oil samples collected from different wells within the Esprito Santo sedimentary basin, Brazil. The samples were coded as follows: Sample A, Sample B, Sample C, Sample D, and Sample E. Prior to any measurement, all crude oil samples were subjected to a rigorous dehydration process to remove formation water and dissolved salts, ensuring that the analyzed properties reflected the oil phase and suspended solids exclusively.^15^ Samples were performed according to ASTM standard practice D5854 ^15^ and laboratory tests according to ASTM standard methods: relative density (ASTM D5002-2022);^16^ API degree (ASTM D1250 and ISO 12185);^17,18^ kinematic viscosity (viscosity) (ASTM D445);^19^ and total acid number (TAN) (ASTM D664).^20^ The elemental composition, specifically the concentration of Fe, Ni, and V, was determined using Inductively Coupled Plasma Optical Emission Spectrometry (ICP-OES) and Mass Spectrometry (ICP–MS) following microwave-assisted acid digestion.^21^ The hydrocarbon structural profile of the crude oil samples was assessed by ^1^H Nuclear Magnetic Resonance (^1^H NMR) spectroscopy in order to calculate the relative contributions of aromatic and aliphatic protons. All samples were dissolved in CDCl_3_, which was also used as the external reference for chemical shift calibration. ^1^H NMR spectra were recorded at room temperature using a Bruker Avance III spectrometer operating at a proton Larmor frequency of 500 MHz, corresponding to a magnetic field strength of 11.75 *T*.^22^

To enable the detection of the dilute magnetic fraction, a magnetic concentration procedure was applied to all samples prior to the EPR experiments.^23^ The crude oils were placed in Falcon tubes and positioned adjacent to a permanent Neodymium–Iron–Boron (FeNdB) magnet. The samples were subjected to a magnetic field intensity of approximately 4000 Oe for a sufficient duration (24 h) to overcome the viscous drag of the oil, causing the magnetic particulates to migrate and concentrate.^24^ This concentrated fraction samples were then used for magnetic measurements. EPR spectra were recorded at room temperature by using an X-band spectrometer operating at a microwave frequency of approximately 9.4 GHz. The magnetic field was swept from 0 to 6 kOe to capture the full ferromagnetic resonance line shape. The EPR spectra were fitted using the theoretical model proposed by Tsay et al. (1971).^13^ It simulates the absorption derivative signal for a system of randomly oriented single-domain particles with cubic magnetocrystalline anisotropy, allowing for the extraction of the line width (*W*), the effective g-factor (*g*), and the anisotropy field parameter (*H*_1_), parameters that help to identify the Fe-oxide phases.

## 3 Results and Discussion

The physicochemical analysis revealed significant differences among the samples, and we will focus on the following parameters: API gravity, Fe, Ni, V contents, and C-based phases. Sample A and Sample C both possessed a low API gravity of 19.2, which classified them as heavy oils. These samples also present the highest TAN values at about 1.8 mg of KOH·g^–1^, indicating a high concentration of naphthenic acids.^25^ However, even considering that these samples have identical API gravity, they show strikingly different rheological behaviors, as shown by the results in Table 1.

**Table 1:** Physicochemical Properties and Elemental Composition of the Crude Oil Samples after Dehydration^a^

Properties	results				
	Sample A	Sample B	Sample C	Sample D	Sample E
API gravity at 60 °C	19.2	28.1	19.2	29.4	22.5
density at 20 °C (g·cm^–3^)	0.9353 ± 0.0008	0.8830 ± 0.0009	0.9351 ± 0.0009	0.8757 ± 0.0008	0.9143 ± 0.0008
kinematic viscosity at 20 °C (mm^2^.s^–1^)	616.97	83.56	622.21	31.95	173.01
TAN (mg of KOH·g^–1^)	1.81	0.3704	1.80	0.10	1.2802
^a^Fe (μg g^–1^)	8.3 ± 0.6	3.2 ± 0.1	4.0 ± 0.2	0.9 ± 0.1	2.6 ± 0.1
^a^Ni (μg g^–1^)	5.8 ± 0.3	5.7 ± 0.3	5.9 ± 0.3	3.8 ± 0.2	5.2 ± 0.2
^a^V (μg g^–1^)	11.5 ± 0.6	11.7 ± 0.5	11.3 ± 0.5	9.1 ± 0.4	9.8 ± 0.5

a Limit of quantification of the method: (a) ICP-OES.

Specifically, Samples A and C exhibited the highest viscosities (≈620 mm^2^.s^–1^ at 20 °C), whereas Sample D showed the lowest viscosity (≈32 mm^2^.s^–1^ at 20 °C).^26^ However, the Fe content of Samples A [(8.3 ± 0.6) μg g^–1^] and C [(4.0 ± 0.2) μg g^–1^] was significantly higher than that of Sample D [(0.9 ± 0.1) μg g^–1^]. Sample D also exhibited the lowest total acid number (TAN, 0.10 mg of KOH·g^–1^) and the highest API gravity (29.2) among the crude oil samples studied. Regarding the Ni content in these crude oils, it is important to say that Ni values are in the range of (5.2 ± 0.2) μg g^–1^ to (5.9 ± 0.3) μg g^–1^ for Samples A, B, C, and E. Again, Sample D presented the lowest Ni value of (3.8 ± 0.2) μg g^–1^. The V values range from (9.1 ± 0.4) μg g^–1^ to (11.7 ± 0.5) μg g^–1^ for Samples A–D. The hydrocarbon structural profile of crude oil Samples A–E was investigated by ^1^H NMR spectroscopy to probe the local chemical environments of hydrogen nuclei and to determine the relative contributions of aliphatic and aromatic phases (Figure 1). The ^1^H NMR spectra are dominated by aliphatic protons in the 0.2–1.9 ppm region, with a smaller contribution from aromatic protons between 6.0 and 9.3 ppm. The calculated aromatic-to-aliphatic proton ratios indicate that all samples are predominantly aliphatic, with Sample D exhibiting the highest aliphatic fraction (ratio ≈1:23), consistent with its lower viscosity. Chemical shifts (δ), expressed in parts per million (ppm), were referenced to CDCl_3_.

**Figure 1:**
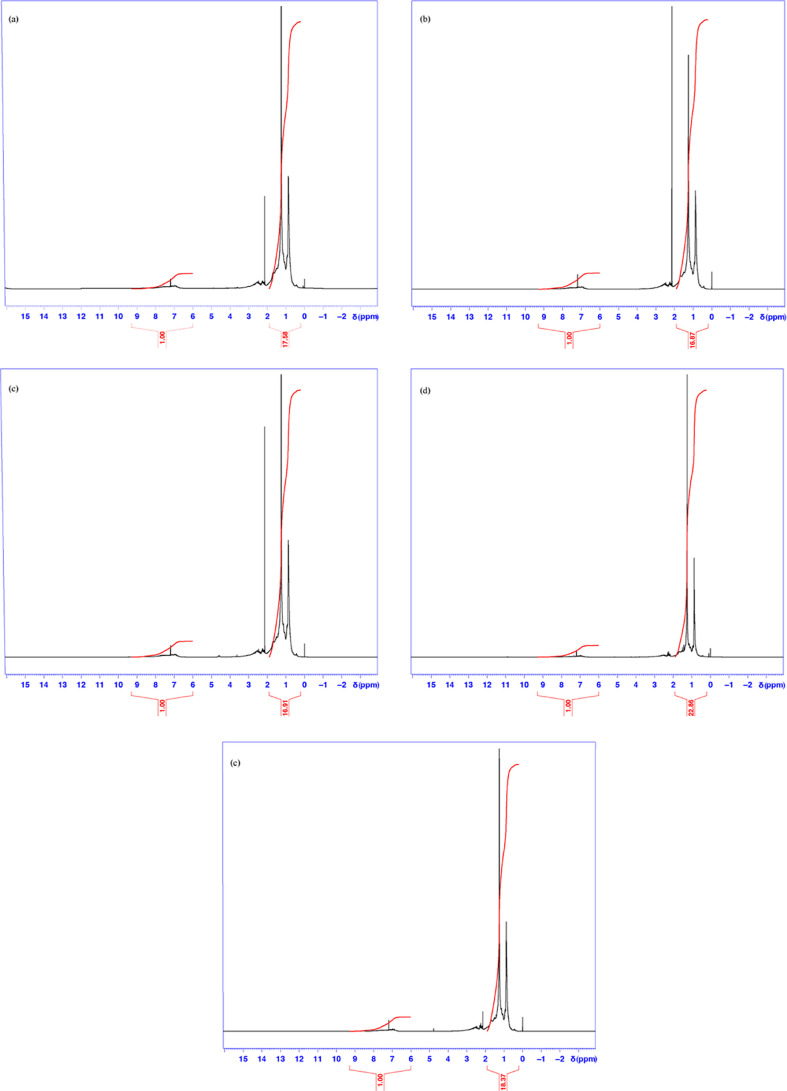
(a–e). ^1^H NMR spectra of Samples A–E recorded at 300 K and referenced to CDCl_3_. The black lines correspond to the experimental data, while the red curves represent the normalized integral traces used to estimate the relative contributions of aliphatic (0.2–1.9 ppm) and aromatic (6.0–9.3 ppm) protons. The red values shown below the chemical shift (δ) axis (horizontal axis) are normalized relative to 1.00.

The elemental analysis provided a crucial context for interpreting the magnetic data. The iron (Fe) concentrations varied by 1 order of magnitude across the samples. Sample A contains the highest iron concentration equal to (8.3 ± 0.6) μg g^–1^, which is strongly correlated to its high acidity (TAN). This association suggests that a significant portion of the iron in Sample A originates from the corrosion of production tubing, where acid attack releases iron ions that subsequently precipitate as oxides.^27^ Conversely, Sample D shows the lowest iron concentration of (0.9 ± 0.1) μg g^–1^. Considering its low acidity and lack of corrosive potential, this iron concentration value represents the baseline for the intrinsic, geogenic iron content in this set of oils.^28^ High concentrations of nickel and vanadium were also found in all samples, as typically found in metalloporphyrins of asphaltic oils.^29^

The EPR spectra for all five samples (A, B, C, D, and E) are displayed in Figure 2.

**Figure 2:**
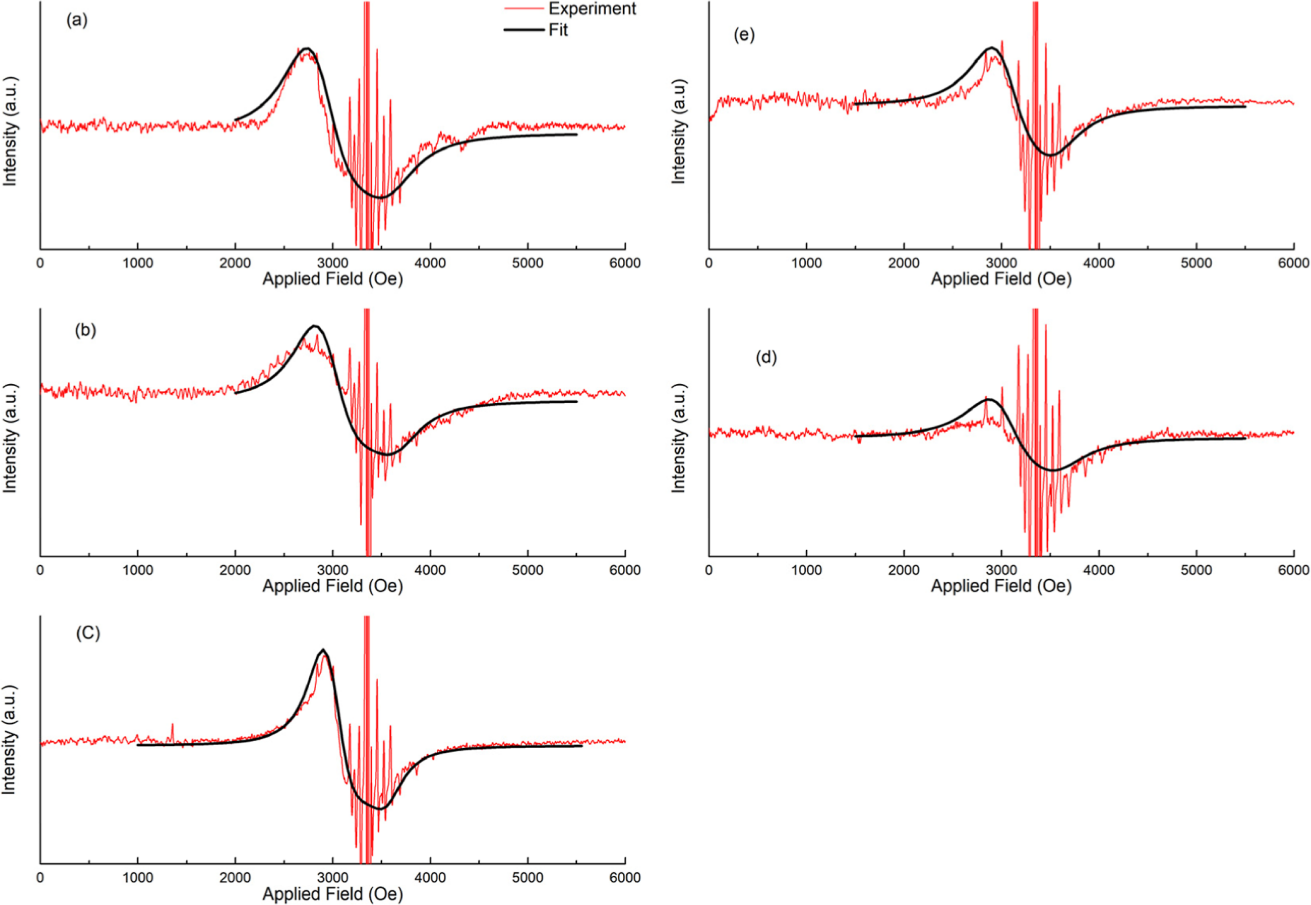
Room-temperature X-band (9.4 GHz) EPR spectra of the five crude oil samples (A–E). Red curves represent the original experimental data obtained in this work, whereas the solid black lines are the theoretical fittings of the broad FMR signal using the mathematical model proposed by Tsay et al.^13^

The experimental data (red curves) reveal a composite signal structure, i.e., FMR and EPR features. First, these spectra are characterized by broad and asymmetric resonance lines, which are typically found in the ferromagnetic resonance of randomly oriented nanoparticle assemblies.^14^ Second, this broad component is superimposed on a set of sharp and multiline hyperfine features characteristic of vanadyl (VO^2+^) porphyrins, which are ubiquitously found in crude oils.^30,31^ One issue that deserves to be mentioned is the absence of sharp hyperfine lines associated with iron, a feature that confirms that the iron signal arises from magnetically ordered clusters (nanoparticles) rather than isolated paramagnetic Fe^3+^ ions. It is important to notice that XRD measurements were also attempted, but due to the low concentrations and nanometric sizes of these particles, no diffraction peak that could be attributed to iron oxides was clearly resolved against the amorphous halo of the oil matrix.^9^ However, the broad FMR line shapes are characteristic of Fe-oxide nanoparticles, specifically maghemite (γ-Fe_2_O_3_).

To quantitatively characterize these Fe-oxide MNPs, the broad component of the experimental EPR data was fitted using the Tsay et al. (1971) model.^13^ The fittings, shown as solid black lines in Figure 2, demonstrate excellent agreement with the broad envelope of the experimental data, successfully isolating the nanoparticle contribution from the vanadyl signal (sharp peaks). The magnetic parameters extracted from these fittings, i.e., line width (*W*), g-factor (*g*), and anisotropy field (*H*_1_), are summarized in Table 2.

**Table 2:** Magnetic Parameters (*W*, *g*, and *H*_1_) Obtained by Fitting the Original Experimental EPR Spectra of the Crude Oil Samples Using the Mathematical Model Described by Tsay et al.^13^

sample	*W* (Oe)	*g*-factor	*H*_1_ (Oe)
A	210	2.15	–480
B	190	2.10	–480
C	130	2.10	–380
D	220	2.08	–380
E	200	2.08	–350

We have first to stress that, in the nanoscale regime, the γ-Fe_2_O_3_ (maghemite) and Fe_3_O_4_ (magnetite) phases are hardly distinguishable by XRD, and even by zero-field ^57^Fe Mössbauer spectroscopy, another powerful method that can be easily used to identify Fe-based species. Similar features are also expected for room-temperature EPR experiments, which alone would not uniquely distinguish the mentioned phases in the nanoscale regime. However, there are some additional issues that may point to a specific Fe-oxide phase under certain assumptions. With this in mind, we begin by pointing out that the effective g-factors obtained for all samples range from 2.08 to 2.15. These g-values are shifted from the free-electron value (*g* ≈ 2.0023) and are consistent with values reported in the literature for γ-Fe_2_O_3_ nanoparticles. In addition, it is known that *g*-values in the 2.05–2.20 range are commonly observed due to magnetocrystalline anisotropy and finite size effects.^32^ Importantly, these values differ from those typically reported for magnetite (*g* ≈ 2.12–2.30 often with a positive anisotropy) or hematite (which exhibits distinct low-field absorption), strengthening the assignment of maghemite as the dominant magnetic phase.^33^ The *H*_1_ field was found to be negative for all samples (*H*_1_ ≈ −350 to −480 Oe). In the context of the Tsay model for cubic crystals, a negative *H*_1_ implies a negative first-order magnetocrystalline anisotropy constant (*K*_1_), which dictates that the easy axis of magnetization lies along the ⟨111⟩ crystallographic direction. This is a known property of spinel ferrites like γ-Fe_2_O_3_,^33^ a phase much more stable than Fe_3_O_4_, the former (γ-Fe_2_O_3_) appears from the oxidation of the latter (Fe_3_O_4_).

The variation in the *W* and *H*_1_ fields across the samples provides insight into the nature of the particles. More specifically, Sample A exhibits the highest magnitude of |*H*_1_| = 480Oe and a relatively high g-factor equal to 2.15. Thus, in combination with its high iron content and high TAN, these properties support the hypothesis of corrosion-derived nanoparticles for this sample. Corrosion products formed in acidic environments often crystallize as maghemite or magnetite with higher crystallinity and potentially larger particle sizes compared to natural geological inclusions, leading to stronger anisotropy fields.^34^ In contrast, Sample D exhibits the largest line width (*W* = 220 Oe) despite having the lowest iron concentration. This broad line width is likely indicative of a wide particle size distribution (polydispersity) and significant inhomogeneity, which is characteristic of natural, geogenic nanoparticles formed through geological weathering or biogenic processes over geological time scales. The low iron content further supports the idea that these particles are intrinsic to the reservoir fluid and not artifacts of production corrosion.^35–37^

While Samples A and D represent the extremes of the corrosion–geogenic spectrum, the analysis of the intermediate samples (B, C, and E) provides further validation of the sensitivity of this magnetic characterization methodology. Sample C, for instance, shares a high total acid number (1.80 mg of KOH·g^–1^) and high viscosity with Sample A yet exhibits the narrowest line width (*W* = 130 Oe) and a lower anisotropy field magnitude (|*H*_1_| = 380Oe). This suggests that while high acidity facilitates the release of iron, the specific crystallographic formation, aggregation state, or domain size of the resulting maghemite nanoparticles is modulated by the rheological environment of the oil matrix, leading to a more homogeneous magnetic population in Sample C. Similarly, Samples B and E display intermediate magnetic parameters (*g* ≈ 2.08–2.10) that do not strictly adhere to a simple linear correlation with iron content, highlighting the complexity of the magnetic mineralogy in mixed-origin fluids. Collectively, these results demonstrate that the combination of the methodology of magnetic concentration and the Tsay model analysis serves as a robust generalized methodology for crude oil fingerprinting. It goes beyond simple elemental quantification, offering a unique “magnetic signature” that reflects the intricate interplay between chemical corrosivity, rheology, and geological history for any given crude oil sample.

It is important to address the challenges and limitations of the developed methodology for detecting iron-oxide nanoparticles in crude oils. The primary challenge lies in the extremely low concentration of natural magnetic nanoparticles in crude oil (often <10 ppm), which prevents detection by standard structural techniques like XRD and/or is hardly measured by ^57^Fe Mössbauer spectroscopy and transmission electron microscopy (we tested these two last methods without success in our samples). Consequently, the magnetic concentration step using a high-gradient field is mandatory; without it, the broad FMR signal of the nanoparticles is often masked by the intense vanadyl hyperfine lines (the Fe-oxide FMR signal is dispersed in the vanadyl background signal, as previously discussed^12^). Furthermore, the phase identification relies heavily on theoretical modeling of the EPR line shape (Tsay model) rather than direct Bragg diffraction peaks. While the fitted parameters (*W*, *g*, and *H*_1_) provide a robust magnetic fingerprint consistent with maghemite, this approach requires careful fitting constraints to distinguish between similar iron-oxide-based phases. In addition, a more systematic study correlating the fraction of Fe content obtained by high-gradient field methodology with the FMR signal should be done to have a full fraction correlation.

## 4 Conclusion

This study successfully demonstrated the detection and magnetic characterization of natural iron oxide nanoparticles that could be often found in crude oils. Specifically, using a magnetic concentration procedure in five crude oils and using EPR spectroscopy, the Fe-oxide-based nanoparticles were identified, and their possible sources were pointed out. Despite the absence of XRD peaks in the five crude oils due to their low Fe-oxide nanoparticle concentrations, the application of the Tsay et al. (1971) model allowed for the positive identification of the magnetic phase as maghemite (γ-Fe_2_O_3_). The extracted magnetic parameters (*W*, *g*, and *H*_1_) revealed distinct differences between the five crude oil samples. A correlation between high TAN, high iron content, and high anisotropy field in Sample A points to an anthropogenic, corrosion-related origin for the magnetic particles. Conversely, the low iron content and broad line width observed in Sample D suggest a natural Fe-species, geogenic origin. These findings highlight the utility of the Tsaỳs model in analyzing the complex FMR spectra of ferrofluids and establish EPR as a powerful, nondestructive tool for fingerprinting magnetic mineralogy in petroleum engineering. Therefore, the magnetic concentration and EPR-based methodology described herein represents an innovative, sustainable, and practical alternative for determining nanoparticles in crude oil samples, emerging as a promising tool for studying the implications of these inclusions in primary oil processing.

## #

P.S.M. Catholic University of Brasília, Brasília, 70790-160, Brazil, and International Center for Physics, Institute of Physics, University of Brasília, Brasília 70910-900, Brazil
